# Psychological therapy for the prevention of suicide in prison: study protocol for a randomised controlled trial

**DOI:** 10.1186/s12888-024-06320-y

**Published:** 2024-12-18

**Authors:** Daniel Pratt, Tim Kirkpatrick, Yvonne Awenat, Caroline Hendricks, Amanda Perry, Leslie-Anne Carter, Rebecca Crook, Paula Duxbury, Charlotte Lennox, Sarah Knowles, Helen Brooks, Linda Davies, Gemma Shields, David Honeywell, Louis Appleby, Patricia Gooding, Dawn Edge, Richard Emsley, Jenny Shaw, Gillian Haddock

**Affiliations:** 1 School of Health Sciences, Faculty of Biology, Medicine and Health, University of Manchester, Manchester, UK; 2 Manchester Academic Health Science Centre, Manchester, UK; 3 Suicide Risk and Safety Research Unit, Greater Manchester Mental Health NHS Foundation Trust, Manchester, UK; 4 Mental Health and Addiction Research Group, Health Sciences, University of York, York, UK; 5 Department of Public Health, Institute for Population Health, University of Liverpool, Policy & Systems, Liverpool, UK; 6 Centre for Reviews and Dissemination, University of York, York, UK; 7 Division of Nursing, Midwifery and Social Work, School of Health Sciences, Manchester Academic Health Science Centre, University of Manchester, Manchester, UK; 8 School of Criminal Justice, Arden University, Coventry, UK; 9 Department of Biostatistics and Health Informatics, Institute of Psychiatry, King’s College London, Psychology & Neuroscience, London, UK

**Keywords:** Suicide, Suicidal thoughts and behaviours, Psychological interventions, Cognitive therapy, Prisoners, Randomised controlled trial

## Abstract

**Background:**

Suicide is the leading cause of preventable death in prisons. Deaths from suicide in prison are significantly, and persistently, elevated compared to those living in the community. Psychological therapies have been shown to be a potentially effective means of alleviating suicidal thoughts, plans and behaviours, but patients located in prison often have no access to evidence-based psychological interventions targeting suicide. The objectives of this programme of research are to investigate the clinical and cost effectiveness of a new psychological therapy programme delivered to male prisoners at risk of suicide.

**Methods:**

The PROSPECT trial is a two-armed single blind, pragmatic, randomised controlled trial and will recruit a target sample size of 360 male prisoners, identified as at-risk of suicide, across 4 prisons in the North of England. Participants will be randomised to receive a psychological talking therapy (Cognitive Behavioural Suicide Prevention, CBSP) plus treatment as usual, or treatment as usual alone. Co-primary outcomes (Suicide Ideation and Suicide Behaviours), as well as related secondary outcomes, will be assessed at baseline and at 6-months follow-up. An intention to treat analysis will be conducted with primary stratification based on prison site and lifetime history of suicide attempt (yes/no). A nested qualitative process evaluation will investigate the nature and context in which the intervention is delivered, with specific focus upon the facilitators and barriers to the implementation of the therapy within prisons.

**Discussion:**

The key outputs from this trial will be to determine whether a psychological therapy for suicidal prisoners is clinically and cost effective; and to generate a project implementation platform that identifies how best to implement the new intervention across the broader prison estate.

**Trial registration:**

ISRCTN (reference ISRCTN14056534 https://www.isrctn.com/ISRCTN14056534; 24th September 2021). Registration confirmed prior to participant recruitment commencing. Modifications to protocol are listed on the study website at ISRCTN.

**Supplementary Information:**

The online version contains supplementary material available at 10.1186/s12888-024-06320-y.

## Background

The World Health Organisation estimate that more than 720,000 people die by suicide every year, and there are as many as 30 times more who attempt suicide [40]. During the last thirty years, the total number of deaths from suicide across the world has persistently increased by over 6% [63]. Within England and Wales, approximately 6000 suicides are registered every year [69], which corresponds to a suicide rate of 11 per 100,000 people. Suicide is the fifth leading cause of death amongst men and the third leading cause of death among 15–29 year olds [94], and yet suicide is preventable.

The problem to be addressed in the proposed programme of research is suicidal behaviour, including suicide attempts, as this has serious psychological consequences including increased risk of subsequent suicide behaviour and death [6, 9]. Already elevated relative to community samples, the rate of suicide behaviour in prison has doubled within the last 10 years. The number of suicidal behaviours in prison reached an exceptional high of 70,875 incidents in the 12 months to December 2023, up 20% from the previous year [60]. Death from suicide is six times more likely in prison [30] with previous suicide behaviour associated with an eightfold increase in risk [29].

The medical and economic costs of suicidal behaviour are profound, with an esimated total cost of suicide to the economy across the UK in 2022 estimated to be £9.58billion [78]. Nevertheless, this cost can seem small in comparison to the ‘intangible costs’,the grief, anger and abandonment of family and friends, and the lost potential of lives cut short (Harris Review, 2015; [53]). This issue has become particularly pertinent to the UK National Health Service (NHS) following the transfer of responsibility for prison healthcare services to the NHS in April 2006. Prisons, working with their NHS commissioned partners, are responsible for protecting the health and safety of their inmate populations and the failure to do so attracts considerable media and political interest, and can be open to legal challenge (e.g. [82]). Compared to community patients, prisoners often present with a greater complexity of needs, including mental health problems, harmful risky behaviours, and socio-economic adversity and exclusion. Prisons predominately incarcerate the most marginalised and deprived people in our society [61].

The development of new evidence-based, suicide behaviour preventive interventions in prisons is highlighted as a priority within UK national suicide prevention strategies [20, 21] and yet the problem has only worsened over recent years. This is in stark contrast to the increasing public and social concern over prisoner suicides. (e.g. [72]). Improving the health of individual prisoners has a number of broader public health implications. The most important of these is that prisoners are often from, and return to, marginalised populations that have poor access to healthcare in the community. Prisoners and their families are a significant part of the socially excluded population [79, 92]. Periods of incarceration, therefore, offer important opportunities for treatment interventions. Taking advantage of this opportunity stands to benefit not only the prisoners themselves, but also the larger public health arena as well as producing downstream savings in other publicly funded services.

International and national policies emphasise imprisonment as offering significant potential to address the pre-existing unmet health needs of this ‘hard to reach’ sector of society and enhance access to interventions aiming to reduce the risk of suicidal behaviour (DH, 2007; [50], [93]. While HM Prison and Probation Service, working with the NHS, has implemented a comprehensive, multifaceted suicide prevention approach, it has yet to systematically deliver any evidence-based psychotherapies targeting suicide behaviour, an omission due largely to the dearth of evidence-based therapies.

When developing new guidelines for mental health provision to adults in contact with the UK criminal justice system (NG66), the National Institute for Health and Care Excellence (NICE) could identify only one previous treatment study, from across the global literature, focussed upon the prevention of suicide behaviour in prison, which was judged to be of very low quality. As such, no recommendations could be made for how prisoners at risk of suicide should best be treated, and the development of a better understanding of and preventative treatment for suicide behaviour within prisons have been called for [66]. These guidelines emphasise that the provision of effective psychological interventions for the management of mental health problems and suicide behaviour is likely to improve survival and quality of life for prisoners. If left untreated, these problems were considered likely to get worse and require subsequent treatment in more resource-intensive settings, such as secondary care or require expensive crisis care. Also, once released back into the community, untreated service users are likely to have repeat interface with the criminal justice system and healthcare services, because their problems are likely to be getting even worse. All of the above are likely to result in a significant increase in healthcare, social care and criminal justice sector costs [66].

Improving access to psychological treatments is a UK government priority with some psychological approaches particularly effective in the reduction of suicide behaviour. In their meta-analysis, Tarrier, Taylor and Gooding [84] found psychological therapy directly targeting suicide behaviour, relative to usual treatment, had an overall positive effect on suicidal behaviours (combined Hedge’s g = –0.59, *p* < 0.001, 95%CI: –0.81 to –0.37). However, this evidence was restricted to community patients and there are substantial differences between community and prison environments. Social climates in prison are characterised by a necessary focus upon security (rather than care), with restrictive treatments delivered by under-resourced healthcare teams amidst a prisoner culture where help-seeking is seen as vulnerability to be exploited (Pratt et al., 2016). Prisoners have limited access to psychological therapies targeting suicidal behaviour often due to organisational or professional boundaries. Prison and healthcare staff may not believe that they have the necessary skills to deal with the needs of suicidal prisoners and therefore may not be willing to offer treatment. Ex-offenders have expressed a strong desire for more ‘taking therapies’ for vulnerable prisoners, with a preference for a pragmatic, solution oriented approach to the use of emotional regulation techniques and overcoming suicidal thoughts and behaviours – views much aligned with the academic literature [48, 68, 71, 75].

Psychological therapies can be criticised for describing a range of principles and techniques, only some of which are optimal in reducing suicidal behaviour. We have addressed this issue by developing a theoretically based psychological model of suicide behaviour that specifies the precise mechanisms to target within a suicide prevention psychotherapy [34, 47, 73]. Informed by this theoretical and empirical work, we have developed and manualised an individualised talking therapy intervention, Cognitive Behavioural Suicide Prevention (CBSP), which aims to address and amend the key aspects described by our psychological model. The feasibility of delivering CBSP therapy has already been piloted in the community with people experiencing psychosis [83], and mental health inpatients [36].

In order to generate preliminary support for the feasibility of improving access to a psychologically informed suicide behaviour prevention programme for prisoners, a pilot trial of CBSP therapy with a small sample of 62 prisoners at risk of suicide was conducted [74]. This study revealed a substantial proportion of previous suicide behaviour within the sample, with only nine (15%) participants self-reporting no lifetime history of a suicide attempt, whereas 35 (57%) had previously attempted suicide on repeated occasions. In terms of treatment acceptability, of 276 therapy sessions offered to participants, only 16 (5.8%) were refused, with an average of nine sessions attended per participant. It is not uncommon for attrition from prison-based trials to be over 50% (e.g. [8, 70]), nevertheless, with sufficient research staff available to maintain regular contact with participants, we were able to support and maintain participant motivation resulting in an attrition rate of only 30%.

Whilst this small study was not sufficiently powered to provide any definitive comment on efficacy, analyses indicated that, relative to controls, the therapy group tended to engage in fewer suicidal behaviours (treatment effect = −0.72, se = 0.47, 95%CI: −1.71 to 0.09). Whilst not statistically significant, this result provides an indication of the potential promise offered by CBSP therapy.

As recognised within the NICE guidelines for mental health of adults in contact with criminal justice system [66], significant and substantial adaptations to community interventions are required because prisoners experience significantly higher levels of psychiatric morbidity, inferior access to care and worse outcomes than the community [45]. Prison-based interventions need to recognise the contextual stressors of imprisonment and their impact upon this vulnerable group. Ways of working with prisoner patients must be adapted to the needs and abilities of this specific client group. Prisoner treatment programmes have been shown to be most effective and acceptable to patients when there exists open, warm and enthusiastic communication between the staff and prisoner [24]. Nurturing and maintaining prisoners’ motivation and willingness to engage in psychological treatment warrants significant attention, beyond that would be typically required for community samples due to the heightened levels of suspiciousness and hypervigilance that become adaptive within prison settings (Pratt et al., 2016). To establish the necessarily therapeutic relationship between the prisoner and practitioner, a substantial level of trust and emotional investment is required from the prisoner [17]. Prisons can be extremely dangerous places from which there is no exit or escape. Prisoners, therefore, need to learn quickly and become aware of the potential dangers within their environment. They learn to become hypervigilant of any possible indicators of personal threat or danger. In order to maintain personal safety and integrity, this can result in an individual adaptively becoming distrustful of others and suspicious of others' intentions. A hypervigilant, distrusting individual may also benefit from projecting an image of themselves as 'tough' or 'hard' since this reduces the likelihood of them being dominated or exploited by other prisoners [55]. Furthermore, to protect oneself from victimisation from other prisoners, an individual may feel the need to suppress their emotional responses to internal and external environmental events. Some prisoners can become emotionally over-controlled and develop a "prison mask" that is unrevealing and impenetrable in order to ensure they are not seen as weak and vulnerable [37]. Whilst this interpersonal style may be adaptive and helpful to the prisoner in coping with imprisonment, it can present a challenge to both the practitioner and prisoner client aiming to engage in psychological therapy [73].

Studies involving samples of men who have previously attempted suicide have consistently found that establishing the trust and respect of mental health professionals is fundamental to men’s initial and ongoing engagement with healthcare services [48, 71, 75]. Many prisoners have little, if any, previous involvement with health services either in prison or in the community. As such, many prisoners commencing psychological therapy have little idea of what to expect. The approach taken to engaging prisoners in psychological therapy needs to be mindful of this lack of experience. Even the use of vocabulary commonly accepted within community settings may not be appropriate, or even off-putting, to prisoners. We have found the use of the word 'therapy' to be a case in point here. Within our pilot trial [74], we moved to offering prisoners a place on a new 'programme' to help people improve how they cope with suicidal thoughts and feelings whilst in prison. The word 'programme' was more acceptable to prisoners since it was often used to describe a number of other activities available to them, e.g. educational programmes (e.g. literacy and numeracy skills), vocational programmes (e.g. bricklaying, plastering) and offending behaviour programmes (e.g. enhanced thinking skills).

Additionally, given that almost all prisoner suicides are male [60], the gender-specific help-seeking attitudes and behaviours of male prisoners need to be taken into account when providing access to treatments. Male suicide has been linked with conformity to traditional (hegemonic) masculine norms [80]. To be seen as strong, resilient, and in control has been identified as a key practice of westernised masculinity [67]. Experiencing suicidal ideation and the associated psychological and emotional distress can often leave the person feeling weak, powerless and vulnerable. Such problems have been theorised to be ‘‘incompatible’’ with masculine ideals and norms [14, 26], thus maintaining the distressing suicidal experience. Some men have justified their decision to seek help by challenging unhelpful perceptions and reframing what it is to ‘‘be a real man’’ [80]. Asking for help, which is viewed by many male prisoners as a ‘‘feminine’’ behaviour, has to be re-evaluated into a brave, rational and practical decision, necessary to re-establish control over one’s life and safeguard survival [48, 68].

Many prisoners struggle with accurately interpreting and controlling emotions [66], which can limit their ability to meaningfully engage in therapeutic activities [10]. In this context, intense but vague sensations of distress can give rise to dysfunctional and destructive behaviours [88], including suicide behaviours (Levi et al., 2008). Prisoners are prone to emotional hijacking [32] whereby there is a tendency to act impulsively whilst experiencing difficult and conflicting emotions with access to rational behaviour and problem solving becoming problematic [43]. Nurturing improved articulation and management of emotions (i.e. emotional intelligence) may enable an individual to find the words to construct a personal narrative to explain, and then begin to understand, difficult and painful experiences within their life.

The fundamentally important first step of offering dedicated support to cultivate trust in the practitioner and nurture emotional intelligence is missing from existing suicide behaviour prevention interventions developed for community-based patients. Within our pilot trial of CBSP therapy for male prisoners [74], the majority of participants allocated to the treatment arm required substantial ‘pre-intervention support’ in order for the necessary open, warm therapeutic relationship to develop. We identified the need for a further modification to the standard delivery of CBSP therapy. Within the current programme of research, we refined the content and resource required for a ‘preparatory phase’ to support patients to better utilise and therefore respond to the subsequent CBSP phase of therapy. As such, the new PROSPECT therapy programme comprised of (i) a preparatory phase, followed by (ii) a CBSP therapy phase. The preparatory phase focuses upon developing trust between the prisoner and practitioner, promoting motivation for therapeutic engagement and nurturing emotional intelligence. This is followed by the delivery of CBSP therapy driven by a theoretically-informed formulation of the patient’s suicide behaviour.

Here we describe a protocol to evaluate the PROSPECT programme, with two important issues to be established. Firstly, we need to know whether the new PROSPECT programme represents a viable first choice treatment for vulnerable prisoners. And secondly, we need to establish the clinical effectiveness of this treatment delivered to a ‘hard-to-reach’ patient group within a challenging environmental context. Demonstrating the effectiveness of the PROSPECT programme for suicidal prisoners has the potential to increase the range of cost-effective treatments for the large number of vulnerable prisoners for whom evidence-based therapies are severely limited [66].

## Objectives

The objectives of this pragmatic randomised controlled trial are to investigate the clinical and cost effectiveness of the manualised PROSPECT therapy programme delivered by trained psychological practitioners to male prisoners at risk of suicide. The primary research question asks “Is the PROSPECT therapy programme more effective than usual treatment for suicidal male prisoners in terms of the hierarchically ranked multiple primary outcomes of (a) suicidal ideation, planning and intent over the last week and (b) the number of occurrences of suicidal behaviour during 6 months from randomisation?” Secondary objectives are to assess (i) whether the PROSPECT therapy programme is more effective on secondary outcomes including: future suicide potential, suicide related cognitions (hopelessness, defeat, entrapment), depressive symptoms, general psychopathology and personality difficulties; (ii) what is the effect of exposure to specific ‘doses’ of the PROSPECT therapy programme where exposure is measured by number of therapy sessions attended, and compliance with treatment; and (iii) is the PROSPECT therapy programme cost effective compared to usual treatment for suicidal male prisoners?

## Methods/Design

### Trial Design

**Fig. 1:**
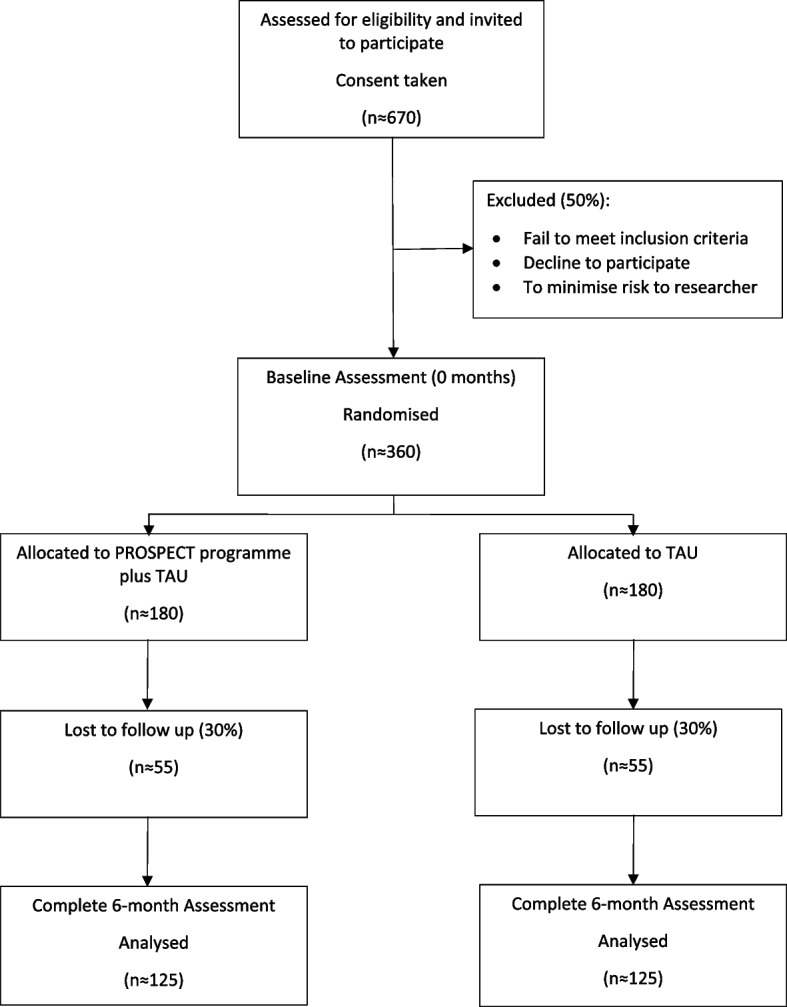
Trial flow chart

The study is a two arm Phase III single-blind randomised controlled trial (RCT) of the PROSPECT therapy programme, in addition to treatment as usual (TAU). To provide opportunity for further iterative development of the PROSPECT therapy programme, we will conduct a 6-month internal feasibility study at the start of the trial. We will evaluate recruitment in months four to six, to allow for a three-month set up period, and expect to be recruiting a minimum of four participants per month, per site. A pause in recruitment will take place to allow the independent Programme Steering Committee (PSC) to assess recruitment rate and to allow for refinement of participant approach and consent process. See Fig. 1 for Trial Flow Chart.

#### Study Setting

The trial will be undertaken within four host prisons located within the UK geographical regions of the Northwest (NW) and Yorkshire and Humber (YH). This trial will be positioned within that part of the prison system where the largest numbers of incidents of suicidal behaviour occur. The majority of incidents of suicidal behaviour are reported for sentenced prisoners for whom two thirds of incidents occur [41].

#### Referrals and Retention of Participants

Within the feasibility trial, the host prison held a capacity of 1238 prisoners, and a referral rate of 12–15 eligible participants per month was achieved [74]. Consequently, to meet the target number of 670 referrals, recruitment will be undertaken in four prison sites whose combined prisoner capacity totals almost 4000 prisoners. To provide assurance that sufficient numbers of eligible prisoners will be available for screening, indications have been provided from each of the host prison sites to quantify the number of prisoners likely to be eligible for screening. Two of the larger prisons (NW1 and YH1) estimated there will be approximately 40–50 prisoners per month at each prison who are eligible, whilst at the smaller prisons (NW2 and YH2), there will be approximately 20–25 prisoners per month at each prison likely eligible for screening. These figures equate to a combined pool of 120–150 eligible prisoners per month across the four prisons. Therefore, a 21-month recruitment period should enable the identification of approximately 360 potential participants meeting eligibility criteria.

Whilst prisoner transfers would be expected to be predictable, an established working practice within many prisons to maintain the security of their establishment is to transfer prisoners around the national estate with very little, if any, prior notice provided to the prisoner. To minimise the impact of this working practice upon this RCT, all participants will consent to being subject to a ‘holding order’, which will require them to remain within the host prison for the duration of their participation in the trial. Also, at the baseline assessment, participants will provide details of how to contact them should they be released during participation in the trial. This may include consent for the researchers to make contact via any organisation (e.g. National Probation Service) the participant may be engaging with. The introduction of Community Rehabilitation Companies [58] will mean that all participants will have supervision in the community and it is anticipated that this will reduce the number of participants lost to follow-up. Whilst the numbers of participants falling into this category are expected to be small due to the eligibility criteria, a post-release follow-up procedure is in place to minimise predictable attrition. Similar follow-up assessments will also be conducted with those participants that are transferred out of the host prison to another prison. Using a similar strategy as described for released participants, the research team will endeavour to make contact with the participant soon after the transfer and update any contact information accordingly. At this contact, the Researcher will arrange a follow-up assessment interview which will take place at the participant’s new prison, where possible.

We recognise that the atmosphere in many prisons can be chaotic due to unpredictable transfers, increased use of seclusion, victimisation, etc. Such real-world issues could affect the recruitment and subsequent follow up of a large sample of prisoners, as proposed by this study. The trial’s recruitment strategy is grounded within an awareness of such a landscape and so the recruitment targets stated are realistically achievable.

#### Eligibility Criteria

The inclusion and exclusion criteria stated below were refined during the feasibility trial [74] to maximise real world applicability to the individuals comprising the prisoner population who are identified to be at risk of suicide and seeking a psychological intervention. The inclusion criteria are (i) person sentence to imprisonment in a male prison, (ii) aged 18 years or over, (iii) at current risk of suicide behaviour as indicated by currently being on the host prison’s ACCT system, or on the ACCT system within the four weeks prior to consent, (iv) able to complete a brief battery of self-report measures with breaks if needed, and (v) willing to consent to being subject to a ‘holding order’ to require them to remain within the host prison for the duration of their participation in the trial. The exclusion criteria are (i) planned release within the next 9 months, (ii) insufficient knowledge of English to enable adequate participation in the assessment process, (iii) deemed by prison staff to be too dangerous/elevated risk of harm to the researcher, and (iv) considered by researcher to be lacking capacity to provide informed consent, according to the British Psychological Society’s code of human research ethics.

(http://www.bps.org.uk/sites/default/files/documents/code_of_human_research_ethics.pdf).

#### Participant Recruitment

Previous feasibility trials [74, 83] enabled the development of a recruitment strategy that proved acceptable and effective, which informed the strategy for the current trial. To launch the study at each site, all governor-grade staff will be invited to a brief meeting to talk about the study. Additionally, information about the study, including eligibility criteria, will be distributed to all prison staff via email, team briefings, and other existing dissemination channels deemed appropriate by the host prison governor. In addition to the launch events, the research team will seek to ensure continuing commitment to supporting the trial is maintained by the prison governors. Senior management support for this trial is crucial in it achieving success, and this shall remain a high priority issue for the research team throughout the trial. Regular communication between the prison governors and the Chief / Principal Investigators will be ensured through project briefings and attendance at Senior Management Team meetings at each host prison.

#### Participant identification and initial approach

Each host prison holds a list of prisoners who are, or who have recently been, cared for under the ACCT system [57]. The research team will work with the Safer Custody link person in each of the four prisons to identify potential participants who meet the eligibility criteria. Previous research has highlighted that successful recruitment in prisons tends to be associated with friendly, respectful, face to face approaches made at the cell door by enthusiastic researchers; it is generally unacceptable for prison staff to be used as gatekeepers as this could bias recruitment [51]. However, members of the research team should not have access to confidential information about potential participants prior to obtaining consent. To address this, the research team will be informed of potential participants who have expressed in interest in being approached. However, individual prisons operate differently and this can impact upon how potential participants are approached. Therefore, the research team will work flexibly with the individual prisons to develop an effective and safe protocol for approaching potential participants.

#### Consent and eligibility

After an expression of interest has been received, the researcher will make the initial approach. This approach will be made verbally and then, if interested, the Participant Information Sheet will be supplied as well as an opportunity for further discussion. Potential participants will be offered a minimum of 24 h to consider the information and whether they would like to take part in the study. Showing sensitivity to reduced literacy levels in this population, the researcher will informally gather information about participants’ reading ability from prison staff prior to making the approach. The researcher will offer to read the PIS aloud to the participant, or, when appropriate, the participant can read the PIS themselves. The researcher will check that the potential participant meets the eligibility criteria, including their willingness to be to be subject to a ‘holding order’ to remain in their host prison for the duration of their participation in the trial. After adequate time has been given, all queries have been addressed and the research team is confident that the potential participant understands the trial and all requirements, they will be consented into the trial.

Consent will be taken by a member of the trial team who has completed Good Clinical Practice (GCP) training, is suitably qualified and experienced, and who has been delegated by the PI to undertake this activity (and this delegation is clearly documented on the delegation log). Participants will provide written consent prior to any trial-related procedures being undertaken. If the researcher reads the PIS and consent form to the participant then another member of staff will witness that the full PIS and consent form have been read aloud to the participant. In these instances, there is a space on the consent form for the witness to also sign. The original, wet-ink signed copy of the participant information sheet and consent form(s) should be retained in the Investigator Site File. Copies of the completed form will be given to the participant and the prison GP.

If new safety information results in significant changes in the risk/benefit assessment, the participant information sheet and associated consent form would be reviewed and updated if necessary. If the participant information sheet and consent form are updated, all participants (including those already being treated), would be informed of the new information, given a copy of the revised documents and asked to re-consent to continue in the trial.

The study will involve vulnerable individuals since the sample will be drawn from the prisoner population and some of its participants may have ongoing mental health problems and/or suicidal ideation. The provision of participant informed consent and no sense of coercion is paramount, and the research team involved in this trial will be careful to explain all aspects of the study to participants in order to ensure that they understand what will be asked of them. If the researcher has any doubt over a participant's capacity to consent, the participant will be excluded from the study. Given the unique problems of gaining consent in a custodial setting, extra emphasis will be given to the potential participants' rights to consent/not to consent and also their right to withdraw at any time, without the need to give a reason for doing so, free of any coercion or negative consequences/ access to services or privileges. If participants do withdraw consent to continue in the study, any information already given by the participant will remain part of the research provided they agree. If participants withdraw consent to continue in the study and withdraw consent for their data to be used in the study, then their data will be removed from the final analysis.

#### Patient registration/randomisation procedure

Immediately following consent and completion of baseline assessments, eligible participants will be randomised to one of the two intervention arms (PROSPECT programme plus TAU or TAU alone). A web-based randomisation system will be designed, using the Clinical Trials Unit’s (CTU) bespoke randomisation system. The randomisation system will be created in collaboration with the trial analyst/s and the CI and maintained by the CTU for the duration of the project. It will be hosted on a dedicated server within the CTU. The CI or delegate will request usernames and passwords from the CTU. System access will be strictly restricted through user-specific passwords to the authorised research team members. It is a legal requirement that passwords to the randomisation system are not shared, and that only those authorised to access the system are allowed to do so. If new staff members join the study, a user-specific username and password must be requested via the research team from the CTU and a request for access to be revoked must be requested when staff members leave the project. Study site staff experiencing issues with system access or functionality should contact the CI or delegate (e.g. Trial Manager) in the first instance.

Participant initials and date of birth will be entered on the randomisation system, NHS number, email addressed, participant names and addresses and full postcodes will not be entered into the randomisation system. No data will be entered onto the randomisation system unless a participant has signed a consent form to participate in the trial. Randomisation will be undertaken by recruiting site staff, by authorised staff logging onto the online randomisation system. A full audit trail of data entry will be automatically date and time stamped, alongside information about the user making the entry within the system.

The research team will undertake appropriate reviews of the entered data, in consultation with the project analyst for the purpose of data cleaning. No data can be amended in the system, however the CI or delegate (e.g. Trial Manager) may request the CTU to add notes against individual subject entries to clarify data entry errors. Upon request, the CTU will provide a copy of the final exported dataset to the CI in.csv format and the research team will onward distribute as appropriate.

Randomisation will be at the level of the individual using the method of block randomisation with randomly varying block size stratified by prison site (NW1, NW2, YH1, YH2) and previous suicide attempt (No, Yes).

#### Methods to protect against sources of *bias*

The following measures will be put in place to protect against potential sources of bias. An independent third party will perform the randomisation of participants to the trial arms which protects against allocation bias. Randomisation will take place only when potential participants have consented to participate and baseline data has been collected and data entered onto the eCRF. Researchers performing the assessments will be blind as to the participant’s treatment allocation with precautionary strategies employed to prevent the researcher from becoming unblinded which will include (i) practitioners to consider room use and diary arrangements in light of potential breaks of masking; (ii) participant reminded by researcher not to talk about treatment allocation, and (iii) researcher to be prevented sight of participant’s full clinical notes until final assessments have been completed. In order to maintain the blind, therapy only assessments will be undertaken by the practitioners delivering the intervention and recorded on separate eCRF which will not be accessible or visible to the researchers. Blind breaches and potential blind breaches will be recorded and monitored and where possible another researcher will undertake follow-up assessments in replacement of a researcher that has become unblinded. All variables have been defined prior to the RCT taking place. Intention-to-treat analyses will be used. Participant throughput will be recorded, e.g., reasons potential participants opted not to participate. Reasons why participants dropped out of the trial will be recorded, if available. The CTU will set-up and manage electronic data capture for all RCT data required for analysis. The research team will undertake appropriate reviews of the entered data.

#### Sample size calculation

We use an approach based on a simple t-test for the between group comparison in the primary outcome which is specifically designed to account for differential clustering or partial nesting between the two arms. It is implemented in –clsampsi- in Stata. This approach requires the following assumptions:

- Effect size: We have powered our trial on the basis of clinical superiority compared to treatment as usual, and will conduct our analysis accordingly. We have powered to detect a standardised effect size of 0.36. This equates to an approximate mean difference of 3.9 in the primary outcome (Beck Scale for Suicide Ideation, BSSI) or 60% reduction in SB using estimates derived from our pilot RCT [74].
- Practitioner number: From our planned staffing of 4 practitioners at any one time, we have allowed for 7 practitioners to be used during the course of the whole trial (to account for practitioners leaving and being replaced). This also helps to improve the generalisability of the trial. We allow for a variance in the number of participants seen per practitioner (i.e. that this follows a Poisson distribution).
- Clustering: We account for differential clustering of 7 practitioners over trial duration in treatment plus TAU arm, average number of patients per practitioner of 18 and ICC = 0.01. No prior estimate of the ICC is available, but we consider this a conservative estimate to what is typically found in psychotherapy trials. We will include pre-specified prognostic variables for the outcome in our analysis models to further reduce the ICC. This approach is robust to observed increases in ICC as the number of practitioners (clusters) increases. For the calculation, we consider the control arm as clusters of size 1 with ICC = 0.
- Power: We assume a conservative baseline-endpoint correlation of 0.6 (0.6 in pilot, 95% CI 0.4–0.7), a two-sided significance level of 0.05 and statistical power of 90% with equal allocation to two arms. Given these assumptions, an analysis set of 250 (125 per group) has 80% power to detect an effect size of 0.36. Power will be increased by inclusion of baseline covariates where possible.
- Attrition: Although attrition rates for prison-based trials can be over 50% (e.g. [8, 70]), our pilot trial was sufficiently resourced to enable research staff to maintain regular contact with participants, resulting in an attrition rate of 30%.

To achieve a target sample size of *n* = 250, and accounting for expected attrition of up to 30% [74], a total of 360 participants will be recruited with recruitment split in proportion to the prison capacity of each of the host sites. According to our pilot RCT, we can also estimate 40% of people will decline to take part and a further 10% will be excluded for security reasons, therefore approximately 670 potential participants will be screened to determine eligibility (see Fig. 1).

#### Trial Interventions

The trial interventions described below have been specified according to existing policy and procedure [57] and the original treatment protocols [73],Tarrier et al., 2013). The delivery of the PROSPECT programme components will be regularly monitored using audio recordings of therapy sessions.

##### Treatment As Usual (TAU)

Participants randomised to the TAU group will receive the standard care according to national and local service protocols and guidelines. HM Prison and Probation Service (HMPPS) supports prisoners identified to be at risk of suicidal behaviour using a care-planning system called the Assessment, Care in Custody, and Teamwork (ACCT) system. On any one day, over 2000 prisoners in England & Wales are identified under the ACCT system [59]. Typically, individual prisoners receive a risk assessment when a potential risk to self is first identified by staff, which then informs a risk management plan of how to keep the individual safe (e.g. levels of monitoring and observation). Additionally, a referral may be made to the prison’s Mental Health Team that can offer psychosocial assessments, pharmacological therapies and nursing support. Treatment as usual will also include routine care provided by the prison’s general practitioner. The prison GP will be informed that the participant is taking part in the trial and the nature of the study.

##### The PROSPECT Programme

In addition to TAU, participants randomly allocated to the PROSPECT programme group will also receive access to a targeted psychological therapy for suicidal male prisoners. The PROSPECT programme is a structured, time-limited, manualised psychosocial intervention developed to treat male prisoners experiencing suicidal ideation and behaviour [73, 83]. The focus of the PROSPECT programme is informed by empirical work that has tested a theoretically-informed psychological model of the mechanisms underlying suicidal thoughts and behaviours amongst prisoners [34, 74]. The PROSPECT programme comprises of a preparatory phase, followed by a CBSP phase.

##### Preparatory phase

The preparatory phase focuses upon developing open, warm and enthusiastic communication between the practitioner and participant, a key requirement for the necessary trusting therapeutic relationship. Supplemented by self-help resources, the practitioner will encourage the participant to reflect upon their motivation for engagement in the PROSPECT programme and clearly delineate preferred outcomes following therapy. The practitioner will also support the participant to develop an understanding of their previous experiences of suicide ideation and behaviour and collaboratively formulate key areas for intervention.

##### CBSP phase

According to our Schematic Appraisals Model of Suicide (SAMS; [47]), the CBSP phase directly targets (i) information processing biases, (ii) appraisals, and (iii) a suicide schema, since these are the psychological processes that trigger and maintain suicidal thoughts and behaviour. Additionally, CBSP has demonstrable potential to address the interactions between clients’ imported vulnerabilities and the toxic prison environment that can culminate in suicidal thinking and behaviours. CBSP draws from established clinical techniques to restructure the three aspects of the SAMS model, including the use of techniques to encourage participants to evaluate appraisals of themselves, their situation and their future, as well as the use of behavioural techniques to identify and rehearse more helpful responses to distressing situations.

##### Duration of the PROSPECT Programme

**Fig. 2:**
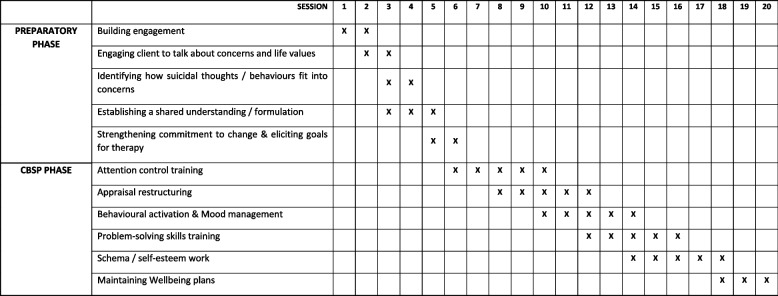
PROSPECT Programme Session Planner

Commencing after randomisation, delivery of the PROSPECT programme consists of up to 20 therapy sessions, delivered across a 6-month therapy window, with each 1:1 therapy session typically lasting 30 to 60 min. As shown in the PROSPECT Therapy Session Planner (see Fig. 2), the initial preparatory phase takes place over the first 6 sessions, with the CBSP phase delivered from session 6 through to session 20. This session planner should only be seen as a guide for the practitioner, with differences expected across patients as delivery is tailored according to individual needs. Nonetheless, specific sessions will be dedicated to the required tasks/techniques within both the preparatory phase and the CBSP phase. For example, within the CBSP phase, up to 5 sessions are assigned to the delivery of each specific therapeutic technique (e.g. appraisal restructuring). This is in accordance with the evidence base for each technique listed. The session planner recognises the opportunity to deliver multiple therapeutic techniques within a given session thus offering a more comprehensive targeting of the psychological architecture behind the patient’s suicidal thinking. However, this approach also presents a limiting factor since an excessive number of techniques being delivered within a single session is likely to undermine the focus of the session, confuse the patient, and so reduce potential for benefit. As such, we have learned to limit the concurrent delivery of therapeutic techniques to no more than 3 techniques within a single session. This delivery format for the PROSPECT programme is consistent with our previous and ongoing trials of psychological therapy to suicidal patients, within inpatient and community settings [33, 36, 83].

### Drop-out from trial intervention criteria

Participants can choose to stop receiving the therapy or they can be withdrawn from the therapy by the trial therapists. In the case of the latter, trial therapists will consult with the participant and the prison and healthcare staff about continuation in the intervention if there is any deterioration in suicidal risk that is deemed to be related to participation in the research trial. Specific criteria for consideration of participant withdrawal will be:

- Occurrence of suicidal behaviour.
- Hospitalisation due to suicidal crisis.
- Clinically significant increase in frequency or intensity of suicidal ideation.

Contingency plans for participants fulfilling any of the above criteria are in line with normal good clinical practice and NICE guidelines [64]. However, if either participant preference and/or clinical judgement indicate a need for participant ‘drop-out’ from treatment for alternative interventions, these will be pursued.

We note that some participants allocated to the PROSPECT programme arm may exercise their right to withdraw from the intervention whilst remaining a participant of the trial. The reason for drop-out will be requested and recorded in the eCRF but the participants’ rights to not state a reason will be respected. Individuals who drop-out of the treatment but remain participants within the trial, will be invited to complete the 6-month follow-up assessment. Such participants will be identified as ‘non-completers’ and distinguished from those who complete treatment (‘completers’) with further sensitivity analyses conducted accordingly.

### Elective discontinuation from the trial by individual participants

Discontinuation from the trial can be decided upon by individual participants. As stated in the consent forms, deciding to no longer participate results in no detriment to participants. Reasons for discontinuing in a trial, as decided by participants, are varied. For example, participants may find that they are no longer willing to engage in the research activities (e.g. researcher interviews, therapy sessions) or have other family or work commitments which they would prefer to prioritise attending. The reason for discontinuation will be requested and recorded in the eCRF but the individual’s rights to not state a reason will be respected. The characteristics of the participants who withdraw or are lost to follow-up will be compared with those that remain within the trial and a sensitivity analysis will be conducted to assess the effects of any missing data.

### End of trial

The trial will end after the last patient recruited at baseline has completed the 6 month (window of 5–9 months) follow up. The declaration of end of trial will be submitted to REC within 90 days of its completion. Following this, all sites will be advised on the process for closing the trial at sites.

### Outcome measures

#### Co-primary outcomes

There are two co-primary outcomes for the trial. The first outcome is a measure of suicidal ideation, planning and intent over the last week. This will be assessed using the *Beck Scale for Suicide Ideation* (BSSI: [3]). This self-report questionnaire has been used extensively in clinical samples, including prisoner patients [74, 89]. The second co-primary outcome is the number of occurrences of suicidal behaviour (SB) during the 6 months from randomisation. Instances of suicidal behaviour will be collected via self-report from participants with researchers using an adapted version of the *Suicide Attempt – Self-Injury Interview* (SASI: [52]) to improve recall and reporting of SB. Following the EME guidance for multiplicity issues in clinical trials, these two primary outcomes are ranked in terms of clinical importance – BSSI, followed by SB.

### Secondary suicide outcomes

Other important outcomes will also be assessed with all such secondary outcome established as highly correlated with suicide behaviour:

- The *Suicide Probability Scale* (SPS: [16]) will be used to assess future suicide potential. The 36-item SPS was developed to measure four dimensions of suicide experience: hopelessness, suicide ideation, negative self-evaluation, and hostility. In a sample of mental health inpatients, the SPS demonstrated high internal reliability with a Cronbach’s alpha of 0.92 (Bisconer and Gross, 2007).
- The *Brief Suicide Cognitions Scale* (B-SCS; [77]) provides a measure of thoughts, perceptions, and beliefs that are commonly experienced by people who have attempted suicide. A Cronbach’s alpha coefficient of 0.91 has been reported for psychiatric inpatients indicating good internal consistency, coupled with a test–retest reliability coefficient of 0.84 [77].

### Mechanistic and other clinical outcomes

We will also assess additional outcomes that have proven to be established correlates and predictors of suicide ideation and behaviour:

- The *Beck Depression Inventory-II* (BDI-II; [4]) will be used to assess depressive symptoms. One of the most widely used measures of depression, this 21-item measure assesses the DSM diagnostic criteria for major depressive episodes. The BDI-II has consistently demonstrated good internal consistency and test–retest reliability amongst adult clinical outpatients [35] and inpatients [81].
- The *Beck Hopelessness Scale* (BHS; [2]) will provide a measure of negative future perceptions. Reliability estimates for the BHS have been reported as 0.88 across various clinical samples, including those with suicidal thoughts and behaviours and severe mental health problems [2].
- The *Defeat and Entrapment scales* developed by Gilbert and Allan [31] measure perceptions of being defeated and trapped, which have been established as key determinants of suicidal ideation and behaviour [86]. Cronbach’s alpha for the Defeat and Entrapment scales were reported as ranging from 0.93 to 0.86, respectively, suggesting high levels of internal consistency [31].

Other important clinical outcomes to be measured are distressing psychiatric symptoms, personality dysfunction, self-esteem and coping. These will be measured through the *Brief Symptom Inventory* (BSI; [22]), the *Standardised Assessment of Personality – Abbreviated Scale* (SAPAS; [62]), the *Robson Self Concept Questionnaire* (Robson, 1989) and the *Coping Inventory for Stressful Situations* (CISS; [27]). A global assessment of wellbeing come from completion of the *ICEpop CAPability measure for Adults* (ICECAP; [1]).

### Health economics measures:

Health status and quality adjusted life years (QALYs) will be measured using the 5-level version of the *EQ-5D* (EQ-5D-5L) and associated published utility tariffs recommended by NICE at the time of analysis [23, 42, 44]. The QALY will be used as the primary measure of health benefit for the economic evaluation. The EQ-5D is a validated generic health status measure, used in national health surveys in the United Kingdom and in clinical trials in mental health, covering five domains (mobility, self-care, usual activity, pain/distress, and anxiety/depression). The five level version will be used in this trial (no problems, slight problems, some problems, severe problems or unable to do activity). The QALY and the EQ-5D are the measures recommended for economic evaluations by NICE [65]. There are 3126 possible health states in the EQ-5D-5L (5 dimensions to the power of 5 levels = 55 = 3126). The health status profiles will be converted to utility values using the published utility tariffs for the EQ-5D-5. The QALYs will be estimated as:

$$\mathrm{QALY}=\sum\left(({\mathrm U}_{\mathrm i}+\;{\mathrm U}_{\mathrm i+1}\right)\;/2\;\times\;\left({\mathrm t}_{\mathrm i+1}\;-\;{\mathrm t}_{\mathrm i}\right)$$

where U = utility value and t = number of days between assessments.

Data about the health and social care services used will be collected from an adapted version of the *Client Service Receipt Inventory* (CSRI; [5]) used in previous and ongoing trials in this population group. All contacts with health, social care, education and third sector organisations are recorded. This measure is designed to capture a broad range of services that participants engage with whilst in prison and following release from prison. For each contact, the table captures the name of service, whether the contact was in prison or the community, the number and duration of contacts, the nature of the contact (e.g. face-to-face, phone call) and who the contact was initiated by. There is good evidence that with support from the person administering the assessment, participants can complete the CSRI to provide valid self-report health service use data [11, 13, 49] with self-reporting shown to be as accurate, valid and reliable as case note review [11].

The cost of the PROSPECT programme will be derived from practitioner time attending initial training and ongoing supervision meetings, as well as a detailed log of therapy provided, completed by the Trial practitioners as part of the fidelity assessment. To maintain the blindness to allocation of research assessors, we will collect data on staff time costs, including training, preparation and clinical supervision, directly from the trial practitioners. The unit costs of NHS and social care services will be derived from national average unit cost data published in the NHS reference costs database, unit costs published by the Ministry of Justice and the annual Unit Costs of health and Social Care and the Unit Costs in Criminal Justice, both published by the Personal Social Services Research Unit (PSSRU). The costs of each service used by a participant will be estimated as the total use of a service multiplied by the unit cost of that service. These will be summed to generate a total cost for each participant.

The EQ-5D-5L and health service use data (CSRI) will be collected for all participants at baseline and 6-month assessments. Health economics data (on psychosocial support) will be collected in sealed envelopes (to maintain blindness) at each assessment and detailed process information about the PROSPECT programme will be collated from the practitioners’ files at the 6-month assessments after programme delivery has been completed. A healthcare reception screen should be conducted for all prisoners arriving at reception into prison custody. This screen is usually conducted by a prison nurse and so the consultation rate will be adjusted to provide a more accurate reflection of service use. The adjusted consultation rate will be calculated by reducing the total number of consultations by one for each new reception [54].

### Therapy process measure

For those randomised to the PROSPECT therapy programme arm of the trial, the therapeutic alliance will be assessed using the Working Alliance Inventory – short form (WAI; [39]), completed by both the participant and therapist, after approximately four sessions and again at the end of the therapy. To determine the effect of exposure to the intervention, the therapist will also record (i) the number of sessions attended, (ii) duration of each session attended, and (iii) perceived level of understanding and psychological formulation developed with the participant.

### Demographic, clinical and criminological data

In addition to the outcome measures detailed above, a range of demographic (age, gender, ethnicity), clinical (mental health diagnoses, previous suicide behaviour) and criminological (index offence, sentence length, previous imprisonments) details will also be collected at baseline. The researcher will also complete a contact sheet for each participant, which will include contact numbers and addresses provided by the participant, as well as a list of services they are likely to be in contact with post-release. This sheet will be completed in collaboration with the participant and the participant will sign the form to confirm they give the research team permission to contact them via the relevant services. Furthermore, clinical and prison records will be accessed, subject to participant consent, to collect relevant information that the participant is unable to self-report accurately, e.g. psychiatric diagnoses, offence details, serious incidents of self-injury and violence.

**Fig. 3:**
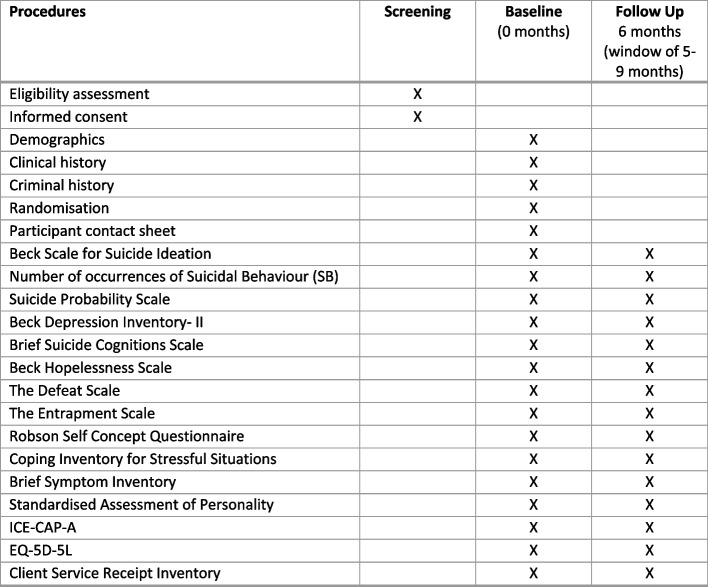
Outcome measures and time-points for collection

See Fig. 3 for further information on which time point each procedure or measure is completed.

We recognise the importance of minimising respondent burden and have worked closely with our service user co-investigator and collaborators in the selection of the clinical outcome questionnaires. We believe that an acceptable assessment pack of outcome measures has been collaboratively developed which takes participant burden into account. We remain open to conducting further rationalisation of this assessment pack, and will prioritise this task within the initial work of the PROSPECT Service User Reference Group (SURG). Whilst a justification can be made for the inclusion of all of the outcome measures, we will continue to liaise with the SURG to reconsider the inclusion of each measure. We also envisage the SURG collaborating with us on the piloting of the administration of the self-report questionnaires to ensure data collection is appropriately conducted in a manner most acceptable to study participants.

### Data Management

#### Data collection tools and source document identification:

A web based electronic data capture (EDC) system will be designed, using the InferMed Macro 4 system. The EDC will be created in collaboration with the trial analyst/s and the CI and maintained by the King’s Clinical Trials Unit for the duration of the project. It will be hosted on a dedicated server within KCL.

The CI or delegate will request usernames and passwords from the KCTU. Database access will be strictly restricted through user-specific passwords to the authorised research team members. It is a legal requirement that passwords to the EDC are not shared, and that only those authorised to access the system are allowed to do so. If new staff members join the study, a user-specific username and password must be requested via the CI or delegate (e.g. Trial Manager) from the KCTU team and a request for access to be revoked must be requested when staff members leave the project. Study site staff experiencing issues with system access or functionality should contact the CI or delegate (e.g. Trial Manager) in the first instance.

Participant initials and date of birth will be entered on the EDC, NHS number, email addressed, participant names and addresses and full postcodes will not be entered into the EDC. No data will be entered onto the EDC system unless a participant has signed a consent form to participate in the trial. Source data will be entered by recruiting site staff, typically within 2 days of data collection by authorised staff onto the EDC by going to www.ctu.co.uk and clicking the link to access the MACRO 4 EDC system. A full audit trail of data entry and any subsequent changes to entered data will be automatically date and time stamped, alongside information about the user making the entry/changes within the system. The CI team will undertake appropriate reviews of the entered data, in consultation with the project analyst for the purpose of data cleaning and will request amendments as required. No data will be amended independently of the study site responsible for entering the data. At the end of the trial, the site PI will review all the data for each participant to verify that all the data are complete and correct. At this point, all data can be formally locked for analysis. Upon request, KCTU will provide a copy of the final exported dataset to the CI in.csv format and the CI will onward distribute as appropriate.

### Data storage and retaining study documentation:

All information will be kept strictly confidential and held in accordance with the principles of the Data Protection Act. Trial data will be stored safely and securely on a dedicated server at KCL. Any information about the participant obtained following their consent from their records held at site will be recorded against a participant identification number (pseudonymised format). Audio recordings of therapy sessions and qualitative interviews will be stored securely (indexed by study number only) on an encrypted and password-protected University of Manchester computer. Only the research team will have access to these data. Paper copies of the relevant trial documentation will be stored at the University of Manchester for a minimum of 15–20 years after publication of the trial results. For audit purposes, electronic copies will be retained for the duration advised by the NHS Research Ethics Committee (typically 5 years). We shall then deposit a pseudonymised data set in the databank maintained by the Offender Health Research Network.

### Data analyses of the RCT

Analysis will follow intention-to-treat principles and we will follow the CONSORT statement for non-pharmacological interventions. Treatment effects for patient-level outcomes will be analysed using a linear mixed models with random effects for practitioner and treatment allocation [76], fitted to the 6-month outcome variables.

Within the first six months of the trial, the trial statistician will develop a detailed statistical analysis plan for primary and secondary outcomes, including any sub-group analyses. This plan will be presented to and agreed with the IDMC and PSC prior to the allocation codes being released and commencement of any data analysis. The statistical analysis plan will be submitted for publication within the trial protocol as soon as is feasible and before the completion of data collection.

The following baseline data will be presented to demonstrate the extent of comparability between randomised groups: demographic (age, gender, ethnicity), clinical (mental health diagnoses) and criminological data (index offence, sentence length, previous imprisonments). A consort flow diagram will be used to show participant flow with reasons (where known) for discontinuation.

Primary analysis of suicide ideation (BSSI) measured at 6 months (window 5–9 months) will use a linear mixed model with adjustment for baseline BSSI, stratification criteria and prognostic baseline covariates. The adjusted mean differences will be presented with 95% confidence intervals. BSSI is expected to be positively skewed and so the standard error and confidence interval for the treatment effect will be estimated by applying a bootstrap procedure [25] using the percentiles based on the results of 5000 replications (using the trial participant as the sampling unit). Primary analysis of suicidal behaviours (SB) measured within 6 months from randomisation will use a mixed model for count outcomes. The appropriate model with be chosen depending on the distribution of this variable. Analysis will adjust for baseline BS, stratification criteria and prognostic baseline covariates. The adjusted incident rate ratio will be presented with 95% confidence intervals. Due to the hierarchical ordering of these primary outcomes, no confirmatory claims can be made with regards to treatment benefit on SB unless it has first been demonstrated on BSSI. Secondary outcomes will be assessed using linear mixed models with similar adjustments. Analysis of primary and secondary outcome measures will be by intention to treat and will therefore include outcomes for all randomised participants in the group to which they were allocated, regardless of protocol adherence.

A therapeutic dose response model will be used to assess the effect of time engaged in therapy on treatment benefit. A complier average causal effect (CACE) model will be used to examine the treatment effect in those defined as ‘compliers’. Compliance will be defined from practitioner notes as a participant having started the ‘CBSP Phase’ of therapy. Treatment effect will be estimated within the subgroups defined by whether a participant has a history of suicide (Yes/No). As the study was not powered to investigate subgroup effects, this will be a purely exploratory analysis. All inferential analyses will adjust for stratification factors used in randomisation. Additionally, baseline outcome and prognostic variables will be adjusted for to improve power. Where outcome variables are skewed, bootstrapped confidence intervals will be presented. There will be no interim analysis of outcome data during the trial.

All efforts will be made to obtain outcome data for randomised participants. This will include following up participants who transfer prisons or are released prior to their 6 month follow up assessment. Reasons for withdrawal of consent to follow up with be recorded and tabulated where participants are willing to provide a reason. The primary analysis will include all available data under a missing at random assumption. Sensitivity to missing data mechanisms will be assessed, and if required multiple imputation or inverse probability weighting used to account for missing outcomes.

### Health economic evaluation:

A detailed economics analysis plan will be approved by the PSC prior to analysis of follow up data. The analysis plan will be informed by published literature supplemented with descriptive analysis of pooled (unblinded) baseline data to identify key covariates for imputation and regression models for the follow up data. The methods used to deal with missing follow-up data will be determined according to the extent and pattern of missing data (e.g. multiple imputation, missing indicator or propensity score methods) [28, 90, 91].

**Fig. 4:**
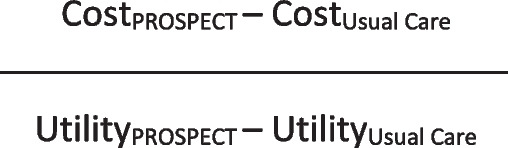
Formula for incremental cost effectiveness ratio (ICER)

The economic data will be analysed using an intent-to-treat approach. The cost perspective is that of the care providers/funders (health and social care and prison services) and the time horizon for the primary care analysis is the 6-month follow up of the trial. The primary measure of interest for the economic analysis is the incremental cost effectiveness ratio (ICER), a joint measure of costs and outcomes. Accordingly, no statistical tests of differences in mean costs or outcomes will be conducted. The ICER is estimated using the formula shown in Fig. 4.

Regression analysis will estimate the net costs and health benefit of the PROSPECT programme, controlling for baseline covariates and used to generate cost-effectiveness acceptability curves (CEAC) for the primary and all sensitivity analyses. Covariates will be determined in the same manner as for the statistical analysis and used to generate cost-effectiveness acceptability curves (CEAC) for the primary and all sensitivity analyses.

The estimates of incremental costs and outcomes from the regression will be bootstrapped to simulate 10,000 pairs of net cost and net outcomes of the intervention group for a cost effectiveness acceptability analysis, as recommended by for health technology appraisals [65]. This will include generating the cost-effectiveness plane, net benefit statistic, cost effectiveness acceptability curves and probability the PROSPECT programme is cost effective compared to usual care [65].

In the absence of a universally agreed threshold willingness to pay value per QALY gained we plan to use a mid-estimate willingness to pay threshold value of £15,000 per QALY gained, with a range of £0 to £30,000 threshold values. The final mid-estimate and range of threshold values will be determined on the basis of published guidance at the time of analysis [12].

Sensitivity analysis will be used to test the impact of assumptions and data on the ICER and results of the cost effectiveness acceptability analysis. These will include: the method of handling missing data, the health benefit measure used in the ICER and the time horizon of the analysis. The choice of sub-groups will be informed by discussion with the clinical team about prison or prisoner characteristics (moderators) likely to affect either costs or health benefit.

There is limited evidence about the effectiveness and cost-effectiveness of suicide prevention programmes in prison settings. Hence, we will use a decision analytic model to extrapolate over a longer time horizon than the 6-month trial-based analysis. The development and analysis of the model will include:

1. A focused review of published evidence and guidelines to identify evidence about:
  - The effectiveness of suicide prevention programmes in prison settings and the maintenance of any effect beyond 6-months.
  - Existing economic models and methods used to evaluate suicide prevention programmes in prison settings.
  - Data about the costs, outcomes and cost-effectiveness of suicide prevention programmes in prison settings.
2. The model structure will be developed iteratively with the full team of researchers and clinical experts. The starting point will be a draft model structure developed from the focussed review and materials developed as part of the research programme.
3. The model will be populated using data from the 6-month trial plus clinical and economic data from the focussed review that allow extrapolation over a longer time period. All the model analyses will use a cost effectiveness acceptability approach to estimate the likelihood that the PROSPECT programme is cost effective.
4. The model will use the 6-month trial data and data from the focussed review to extrapolate the costs, outcomes and cost-effectiveness estimates over longer time frames. We will use assumptions/scenarios about the longer-term impact of the intervention, which will be developed with the full research team. We will use threshold analysis to determine the minimum effect required for the PROSPECT programme to be cost-effective. If there is no observational or trial-based evidence from the review about the likely effectiveness of suicide prevention programmes beyond 6 months, the model will be used to conduct extensive threshold and scenario analyses to assess how effective the PROSPECT programme would need to have more than a 50% likelihood of being cost-effective.

### Process evaluation

Alongside the RCT, there will also be a process evaluation that investigates the nature and context in which the intervention is delivered. Specifically, we will ask “What are the facilitators and barriers to the future implementation of the PROSPECT programme across the prison estate?” The design of the process evaluation follows the MRC guidance on process evaluations of complex interventions. We will draw on a recognised implementation framework, iPARIHS (Integrated Promoting Action on Research Implementation in Health Services, [38], to inform data collection and analysis. The mixed method process evaluation will draw on multiple sources of data to create a rich picture of implementation in practice, enabling us to understand both barriers and facilitators to delivery of the intervention, and to determine if and how implementation success or failure contributes to the RCT outcomes.

Data to inform the different elements of the process evaluation will be collected via semi-structured interviews with staff and prisoner participants, an observational study, and quantitatively with regard to the level of implementation of the Prospect programme. The strategy has been developed to enable the process evaluation research questions to be addressed, but sufficiently flexible to enable the process evaluation to adapt to the needs of the project. A key consideration for the data collection strategy was to ensure a balance was struck between capturing a representative breadth of data and views as well as the depth of information to understand participant journeys through the Prospect programme.

### Semi-structured interviews:

Semi-structured interviews will be conducted with participants receiving the Prospect Programme, practitioners delivering the Prospect Programme, the PROSPECT researchers recruiting participants, and staff employed in key roles within the four host prisons. For prisoner participants, we will interview some participants only once, but others will be interviewed up to three times to understand the barriers and facilitators of participation at key time points in delivery of the Prospect programme. Across the four prisons, we plan to recruit up to 48 prisoner participants who are receiving the Prospect programme. Of these, we anticipate interviewing between six and 24 participants up to three times, depending on the needs of the project. There will be some prioritisation to interview participants during the internal pilot stage to identify any early implementation issues. We will also interview between 8 and 16 staff participants, selected from prison-based staff involved in key roles in the care and management of prisoners who present a risk to themselves. Furthermore, the trial therapists / practitioners directly involved in delivering the Prospect programme (n = 4–10), and those with experience working as a PROSPECT trial researcher (n = 4–10) will also be invited to take part in semi-structured interviews. In order to capture early implementation challenges faced by the practitioners, as well as obtaining a good understanding of how such challenges are resolved or persist throughout the life of the trial, we plan to interview each Prospect Practitioner and Researcher approximately every six months, up to a maximum of four interviews over the two years of the trial.

### Observational study

The purpose of the observations is to learn more about any contextual factors that could act as barriers or facilitators to the implementation of the PROSPECT intervention. In line with the I-PARIHs context construct, relevant contextual factors could include the priorities, culture, and relationships present within the establishments where PROSPECT is being delivered. We will use maximum variation sampling based on the observed characteristics of the professional sample. Across the four prison sites, we will recruit four to eight members of prison staff. Participants for this study will be identified from within the interview sample. The observations will be focused and targeted in nature and aim to explore any tensions/gaps/inconsistencies that emerge from the interview data, as well as relevant contextual factors. The observations will focus on planned activities attended by prison staff or Prospect staff who have taken part in semi-structured interviews. Observed activities MUST relate to the management of suicide or self-harm in prison. These could include (but are not limited to) ACCT reviews, safety intervention meetings, complex case meetings, and mental health case reviews or handover meetings. There can also be an element of snowballing. For example, if during the observations or semi-structured interviews other planned activities that would be beneficial to observe come to light, then these can also be pursued.

### Process evaluation analysis plan

We will produce descriptive statistics for the quantitative outcomes. For the qualitative data, we will combine interview transcripts and field notes recorded from the observation case studies in Nvivo. We will use Reflexive Thematic Analysis, guided by the iPARIHS framework, to create higher order themes. The initial qualitative analysis will be completed blind to the trial outcome to enable open exploration of the themes. We will use a convergence coding matrix to synthesise the different data sources [87]. The synthesis will be interpretative rather than aggregative (as the qualitative and quantitative data cannot be formally combined). We will tabulate the data from each part of the evaluation according to the MRC implementation constructs. This will be in the form of summary statistics for the quantitative data and higher order themes and selectively coded exemplar text from the qualitative data. We will organise data corresponding to each site, to enable us to check for differences and similarities across settings. The analysis team will iteratively review within and across the organised data to identify patterns, guided by the iPARIHS framework (i.e. attending to interacting roles of context, facilitation, recipients and innovation) and reflecting back on the pre-existing prototype logic model [15]. The goal output is to produce a revised “Type 4” logic model [56], whereby the prototype logic model is tested and adapted using the process evaluation data. This enables completion of a contextually sensitive and evidence based final logic model for future delivery of the programme.

### Fidelity measures

In the feasibility trial, a protocol was developed for offering the PROSPECT programme for suicidal prisoners which incorporated checks for patient accessibility/acceptability, practitioner adherence, and therapeutic alliance [74]. Despite prisoners being described as a difficult-to-engage clinical group, over 70% of participants randomised to therapy attended five or more sessions, out of a maximum of 20, with an average of 9 sessions attended. The ‘Did Not Attend’ rate was less than 6%. Ratings of practitioner adherence to the protocol and participant acceptability were above acceptable levels [74].

Within the nested process evaluation, we will assess treatment integrity by monitoring adherence/fidelity to the PROSPECT programme protocol and practitioner competence. Treatment fidelity will be ensured through regular supervision of the trial practitioners and assessed by audio recordings of therapy sessions, where permission to do so has been granted by the participant, with 10% (randomly chosen) rated for adherence to the treatment manual by a member of the project team using the bespoke Treatment Module Checklist, which lists the permitted treatment modules. Also, practitioners and participants will be invited to complete an assessment of the therapeutic alliance [39], after approximately four sessions and again at the end of the therapy.

### Custodians of the data

It should be noted that the Chief Investigator is the custodian of the data.

## Discussion

### Strategies to improve and monitor treatment adherence

The PROSPECT programme will be delivered by accredited Psychological Practitioners (e.g. BABCP accredited Psychological Practitioners or HCPC registered Clinical/Forensic/Counselling Psychologists) with one practitioner allocated to each of the four prisons in the intervention arm of the trial. Trial Therapists / practitioners will already be well trained in delivering psychological therapies, but they will also undergo an intensive training period to gain familiarity with the specifics of the PROSPECT programme, led by the programme team members. The training will involve a combination of interactive lectures and seminars, modelling, role-plays, reading assignments, and homework exercises, which will be delivered by the Chief Investigator (DP) who has extensive training and several years of experience with the CBSP intervention. We anticipate this training will be delivered once at the outset of the trial, although individual training will also be delivered for any replacement practitioners who join the trial at a later date. During the trial, practitioners will receive fortnightly supervision from the Chief Investigator (DP) and other experienced Clinical Psychologists familiar with the treatment protocol. Teleconferencing technologies will be used to ensure practitioners working in both the Northwest and Yorkshire regions can access clinical supervision. Supervision will focus on individual client work, techniques delivered by the therapist, intervention planning and problem-solving any barriers to implementation. Adherence will be monitored throughout the intervention delivery period using a structured therapy delivery diary.

### Ethics, governance and safety

#### Strategies for assessment and management of risk

The safety of participants will be of paramount importance throughout the trial. Whilst some participants may feel some distress whilst completing some of the patient questionnaires, there is no reason to believe that research procedures will increase suicide risk to participants. An established literature has now refuted the idea that discussing suicidal behaviour *automatically* increases the risk of such behaviour [7, 18, 19, 46] with findings suggesting that individuals are more likely to derive benefit from participation than experience harm [85]. Nevertheless, a pertinent ethical issue of this study may be the increased burden for participants in completing the self-report assessments.

From the outset, we will take care to inform participants exactly what is involved in taking part, and allow for as many comfort breaks as needed, with all assessment measures handled sensitively and at the participant’s preferred pace for completion. In a previous study, we found that many participants reported positive experiences of taking part in suicide prevention research, in terms of feeling involved in important research, the cathartic value of talking about suicide, and an enjoyable inter-personal experience [85].

This trial may actually benefit individual participants since cognitive behavioural therapies are not routinely available for suicidal patients in prison. Furthermore, all participants taking part in this trial, in both the PROSPECT therapy programme plus TAU and the TAU alone groups, will receive an enhanced level of monitoring such that any participant deemed to be at suicidal risk will be identified and immediately directed to appropriate care available within the host institution. Our feasibility trial with this patient group reported a modest benefit of entering the TAU condition, likely due to the additional monitoring and sensitive manner of the research assessors [74]. No treatments will be withheld during the trial, with all participants having access to the support they were receiving before the research study (e.g. Prison GP, Mental Health Team, Drug & Alcohol Team), so there is no expected disadvantage in taking part. Participants will not receive payment for participating in the trial, but assurances will be sought from the host prison governor that participants will be authorised to miss their usual work responsibilities in order to attend therapy or research appointments without affecting their usual wage.

### Participant risk management

Inherent in the nature of the population eligible for the trial is the risk of suicide. All participants will remain under the care of the prison GP throughout the duration of the trial. The prison GP will be responsible for all patient-level treatment and management decisions, including prescribing, referral and assessment of risk. This arrangement will be made clear to all clinicians and prison staff involved in the ongoing care of patients in this trial. The pragmatic nature of this trial means that we will not seek to influence this arrangement.

In addition to the usual arrangements described above, participants in the trial will also be subject to our detailed policies and standard operating procedures, informed by good clinical practice, for monitoring and managing suicide risk during all researcher encounters with trial participants. The distress protocol will be followed should any participant disclose suicidal thoughts during any encounter with the research team, or if the research team have reason to suspect the participant is experiencing an increase in suicide risk. Research Assistants (RA) will be well-trained in our distress protocol and receive regular clinical supervision to ensure adherence to the procedure. Participants’ suicide risk status is assessed at each of the trial’s assessment interviews, including follow-ups, with specific questions focussed upon suicidal thoughts and plans. The distress protocol worked effectively in our feasibility trial where no serious adverse reactions were recorded.

### Researcher risk management

All members of the research team, which includes practitioners and RAs, will abide by the lone working policies of their employer(s). For the aspects of the research that take place in a prison setting, the research team will undertake the necessary induction training and security clearances as determined by the individual prison and will abide by the health and safety policies and procedures of the prison. A risk assessment for the study has been conducted and a researcher safety protocol is in place that all research staff will be familiar with prior to undertaking research activities. All researchers will receive clinical supervision on a fortnightly basis and will be able to speak to the CI or Programme/Trial Manager between clinical supervision sessions.

There is a possibility that research participants may tell the researcher information that the researcher will have a duty to disclose to the appropriate authorities. Participants will be informed via the Participant Information Sheets that the researchers may have to disclose any information that raises concerns about participant safety or the safety of others, information regarding undisclosed illegal acts or any current plans of future illegal activities behaviour that is against the prison rules, or information that raises concerns about terrorist, radicalisation or security issues. Additionally, prison staff or other professionals taking part in the research will be informed that researchers will have a duty to inform the appropriate authorities should they disclose information about misconduct or poor practice.

### COVID-19 risk

COVID-19 is likely to be an ongoing, dynamic risk factor throughout the duration of the trial. This includes risk to the research staff, practitioners, and participants in terms of increased risk of getting COVID-10, but may also interfere with recruitment of participants, data collection, and delivery of the PROSPECT programme. Individual prisons have, in collaboration with Her Majesty’s Prison and Probation Service (HMPPS), developed protocols for safe working. These protocols include varying levels of restrictions depending upon the number of cases of COVID-19 within each prison and the level of cases/transmission within the wider geographical area of each prison.

At all times, the research team will adhere to the protocols in place at each of the prisons to minimise risk to the participants, members of the research team, and to the staff working in the prison. This will include the wearing of protective clothing, using meeting rooms with adequate space to allow for social distancing, and potentially limiting contact with participants to remote contact. In the event of the latter of these restrictions, the research team will continue to deliver the PROSPECT programme via phone or tablet as discontinuing the therapy may present a risk to participants who have already started therapy. Research activities, including recruitment and data collection, will continue where possible, although may be paused for a period of time until face-to-face contact is permitted again. This may have implications for the timely completion of the RCT, but as the study is being run in four prisons any restrictions in one prison can be offset by increasing research activity in the other prisons.

### Safety reporting

#### Adverse Events (AEs)

An adverse event (AE) reporting system will be used on the trial. Suicidal ideation, self-harm, harm to others and property will be routinely recorded and it is expected that these will occur for some participants. These will include, ideation alone with no behaviour (suicidal ideation or self-harm ideation), self-harm or suicidal behaviour (cutting self, biting and breaking the skin, severely scratching or running anything on the skin, using corrosive substances on the skin, sticking sharp objects into the skin, pulling out hair, burning with a cigarette lighter or match, scolding the skin, swallowing objects, choking or blocking airways, inserting objects into body cavities, overdosing with prescribed medication, overdosing with non-prescribed medication, taking illicit substances (not including medication), taking poisonous substances, inducing vomiting, over-eating, eating something that is known to cause an allergic reaction, food or fluid refusal, refusing medication, violence towards an inanimate object (e.g. a wall or door), ligaturing/hanging, drowning, severing body parts, electrocuting), events involving others (threats to harm others / violence, actual harm to others / violence). It should be noted that these may be Serious Adverse Events (SAEs) depending on the severity of injuries and outcomes of the behaviour. These events do not require further follow up unless there is evidence that these are research related but each event should be recorded from consent until 1 month after the final follow-up assessment is completed. Any untoward medical occurrence that is not included in the definition above will not be reported.

#### Serious Adverse Events (SAEs)

All SAEs occurring from the time of consent until 1 month after the final follow-up assessment completion will be recorded on the SAE report form and emailed to the Prospect Trial Manager and CI (as sponsor’s representative) immediately and within 24 h of the research staff becoming aware of the event. Any change of condition or other follow-up information will be reported to the Trial Manager and CI as soon as it is available, or at least within 24 h of the information becoming available. Events will be followed up until the event has resolved or a final outcome has been reached.

The Trial Manager and CI will review the SAE form. If the SAE is related to participation in the trial, the CI will review the expectedness in relation to the nature or severity of which is not consistent with the effects or consequences of participation in a psychological intervention trial. All adverse events that are research related will be reported by the CI to the relevant host prison responsible clinical team and the Data Monitoring and Ethics Committee (DMEC) on a regular basis and in an expedited fashion. Fatal or life-threatening events will be reported to the Programme Steering Committee, the DMEC and the relevant Ethics Committee within seven days of knowledge of such cases.

#### Monitoring of adverse events

Due to the nature of the participant sample, it is anticipated that a number of suicide-related adverse events (expression of suicidal ideation, intent, behaviours and attempts) will occur in both the active and control arms of the research trial. As stated, suicidal behaviour is one of the primary outcome measures of this study. However, should the IDMC become aware of an inflated rate of suicide-related adverse events in the active, relative to the control, arm of the trial that is attributable to participation in the trial, then the PSC may be alerted to consider recommending a premature discontinuation of the trial (Duggan et al., 2014).

#### Reporting urgent safety measures

The Sponsor or Investigator may take appropriate urgent safety measures (USMs) in order to protect the participant of a clinical trial against any immediate hazard to their health or safety without prior authorisation from the ethics committee. Where the researcher / practitioner takes urgent action that is not consistent with the protocol to prevent harm to a subject on a trial, they must immediately inform the Trial Manager and give full details of the measures taken and the decision-making process surrounding the action(s) taken. The Trial Manager will inform the CI, Sponsor and REC of these measures immediately, but no later than 3 days from the date the actions were taken. Written notification in the form of a substantial amendment is also required, which is anticipated within approximately 2 weeks of initial notification.

### Dissemination

Established dissemination practices will be adopted with a number of methods of disseminating the findings of the research to be used in order to ensure various target audiences receive feedback from the study. Results will be written up for a peer-reviewed publication in an academic journal. Additional publications will also be sought to reach a wide practitioner audience, rather than a single speciality publication. Findings will also be presented at national and international conferences and/or seminars to other mental health and forensic professionals. Throughout the study, a regular newsletter will be produced and made available to all prisoners and staff, which will summarise the current progress of the study and reinforce participation and support.

To feedback the results of the study to potential recipients of the new PROSPECT programme and other interested users of prison services, a series of presentations and seminars will be held with the local and national offender forums and agencies. We will collaborate with the ex-offender SURG group with such activities. Also, our collaborating charity partners have agreed to promote the study and its findings throughout the service user and offender communities. We shall also use our existing relationships with other prison charities to ensure the findings and implications of the study can be disseminated via forums and publications targeted at prisoner governors and policy makers. A summary of findings will also be forwarded to every prison governor in England and Wales.

Furthermore, as members of the Health and Justice Research Network (https://sites.manchester.ac.uk/hjrn/) with established links with HM Prison and Probation Service’s Safer Custody Group, we will be able to inform the development and delivery of psychological interventions for the prevention of suicide in prisons, both on a local and national level. We will also make recommendations to regulatory and advisory bodies, including NICE, British Psychological Society, and the Royal Colleges of Psychiatry and Nursing.

### Patient and Public Involvement (PPI)

The authors’ programme of prison suicide prevention research has been inspired and informed by several service users who identified an area of unmet need based on their own personal experiences. By working alongside people with lived experience, in a truly collaborative and open manner, we are confident this trial is grounded in the concerns of people who have experienced suicidal crises and distress whilst incarcerated in prison. The research team includes a service user and service user collaborators.

The PPI lead (DH), who has personal experience of suicidal distress and imprisonment, has been fundamental in the planning, decision making, design and development of this trial alongside fellow co-investigators. Working in partnership with the CI, the PPI lead has substantially been involved in carrying out extensive project development work, including reviewing relevant literature, investigation and evaluation of current service provision, advocating offender stakeholder views, and consolidating the formation of the project team to provide the necessary expertise for this research. Additionally, we were awarded a small grant from the Public Involvement Fund of the NIHR Research Design Service North West which enabled the research team to conduct two open events where an overview of the PROSPECT programme was presented to people with lived experiences of suicidality as prisoners. We have also consulted with our existing service-user reference groups that are associated with NIHR and MRC grants investigating suicide in mental health inpatients and people experiencing psychosis. These events and consultations have contributed advice and opinion on the aims, design and methods of the current protocol. One example of a specific change resulting from this work is the employment of a Service User Researcher within the study to help maximise recruitment and engagement of participants in this particularly sensitive mental health issue.

Furthermore, we are working with local and national offender health service user organisations to shape the research design, methods and processes to be adopted in the trial. Specifically, we are working with Revolving Doors to ensure continued consultation and active service user involvement throughout all stages of design and execution of the research development process. These collaborations have enabled us to establish our framework for the PROSPECT Service User Reference Group (SURG). The PROSPECT SURG comprises of reformed offender researchers and local ex-offenders with experience of prison healthcare services, and is chaired by our PPI lead. All members of the SURG will have personal experiences of suicidality within a prison setting, either directly themselves or as Samaritans trained ‘Listeners’ working within the prison. The SURG will lead on the public and patient involvement elements of the study and provide a forum for consultation throughout the duration of the trial.

As established within the feasibility trial, the SURG will be integral to the delivery of the proposed study and supported in maintaining active involvement in all stages of the project from jointly developing the work described in this application through to the recruitment, data interpretation, reporting, and dissemination. Specifically, the Service User Co-applicant will be invited to represent the SURG at all Programme Management Group meetings and members of the SURG will be routinely invited to attend the Programme Steering Committee. As is standard practice for our trials, the selection and writing of participant materials (information leaflets and consent forms, clinical materials, training materials, etc.) will be overseen and edited by the SURG. SURG members will also be influential in research and clinical staff training needs associated with the study and with providing an on-going focus on ethical matters concerning the emotional and practical needs of participants.

The SURG will also be actively involved in developing and delivering our dissemination strategy to ensure the outcomes of the trial are communicated in a manner that is inclusive and available to people who use prison healthcare services. Informed by our feasibility trial dissemination strategy, we will deliver a series of presentations and seminars held with offender forums and agencies, at a local and national level. We follow national guidelines on the involvement of the public in research and our payment procedures for SURG members’ contributions are in keeping with national recommendations (see www.invo.org.uk).

### Trial management

A Programme Steering Committee will be established and will comprise an independent chair, an experienced clinician, an independent statistician, and at least one service user representative with lived experience of the criminal justice system. The Chief Investigators (CI) will also attend the PSC accompanied by other senior study collaborators, where necessary. The PSC will oversee all aspects of the research including the trial and will make decisions on its continuation including the go/stop criteria for the internal pilot. The PSC will meet at least once per year.

An Independent Data Monitoring Committee (IDMC) will be established with all members to be independent as defined by NIHR research governance guidelines and should comprise of a clinician as chair, a further clinician and a mental health statistician. The IDMC will review serious adverse events considered by the CI to be research related and look at outcome data regularly during data collection. The Trial Statistician will attend the IDMC as appropriate. The Chief Investigators will only attend the IDMC when invited by the IDMC Chair. The IDMC will meet at least once per year.

The Programme Management Group (PMG) will consist of the Chief Investigator (DP), co-investigators, Programme/Trial Manager, and Trial Statistician and will consider day-to-day management issues and the overall progress of the trial. The PMG will meet on an at least monthly basis.

## Supplementary Information

Supplementary Material 1.

## Data Availability

No datasets were generated or analysed during the current study.

## Abbreviations

ACCT: Assessment, Care in Custody and Teamwork
AE: Adverse Event
AR: Adverse Reaction
BABCP: British Association of Behavioural and Cognitive Psychotherapies
BDI: Beck Depression Inventory-II
BHS: Beck Hopelessness Scale
B-SCS: Brief Suicide Cognitions Scale
BSI: Brief Symptom Inventory
BSSI: Beck Scale for Suicide Ideation
CBSP: Cognitive Behavioural Suicide Prevention therapy
CI: Chief Investigator
CISS: Coping Inventory for Stressful Situations
CSRI: Client Service Receipt Inventory
CTU: Clinical Trials Unit
DH: Department of Health
DHSC: Department of Health and Social Care (formerly Department of Health, DH)
IDMC: Independent Data Monitoring Committee
eCRF: electronic Case Report Form
EDC: Electronic Data Capture
EQ-5D: EuroQol- 5 Dimension
GCP: Good Clinical Practice
HMPPS: His Majesty’s Prison and Probation Service (formerly His Majesty’s Prison Service, HMPS)
ICECAP: ICEpop CAPability measure for Adults
ICER: Incremental cost effectiveness ratio
iPARIHS: Integrated Promoting Action on Research Implementation in Health Services
KCL: King’s College London
KCTU: King’s Clinical Trials Unit
MAHSC: Manchester Academic Health Science Centre
MRC: Medical Research Council
NHS: National Health Service
MoJ: Ministry of Justice
NIHR: National Institute of Health Research
NICE: National Institute for Health and Care Excellence
NW: Northwest region of England
PGfAR: NIHR Programme Grants for Applied Research
PMG: Programme Management Group
PROSPECT: The prevention of suicide behaviour in prison: Enhancing access to therapy programme
PPI: Patient and Public Involvement
PSC: Programme Steering Committee
QALYs: Quality-Adjusted Life Years
RCT: Randomised Controlled Trial
REC: Research Ethics Committee
SAMS: Schematic Appraisals Model of Suicide
SASI: Suicide Attempt – Self-Injury Interview
SAE: Serious Adverse Events
SD: Standard Deviation
SAP-AS: Standardised Assessment of Personality – Abbreviated Scale
SPS: Suicide Probability Scale
SURG: Service User Reference Group
TAU: Treatment as Usual
WHO: World Health Organisation
YH: Yorkshire and Humber region of England
